# Prediction of Cell Penetrating Peptides by Support Vector Machines

**DOI:** 10.1371/journal.pcbi.1002101

**Published:** 2011-07-14

**Authors:** William S. Sanders, C. Ian Johnston, Susan M. Bridges, Shane C. Burgess, Kenneth O. Willeford

**Affiliations:** 1 Department of Biochemistry and Molecular Biology, Mississippi State University, Mississippi, United States of America; 2 Institute for Genomics, Biocomputing, and Biotechnology, Mississippi State University, Mississippi, United States of America; 3 Department of Basic Sciences, College of Veterinary Medicine, Mississippi State University, Mississippi, United States of America; 4 Department of Computer Science and Engineering, Mississippi State University, Mississippi, United States of America; University of California San Diego, United States of America

## Abstract

Cell penetrating peptides (CPPs) are those peptides that can transverse cell membranes to enter cells. Once inside the cell, different CPPs can localize to different cellular components and perform different roles. Some generate pore-forming complexes resulting in the destruction of cells while others localize to various organelles. Use of machine learning methods to predict potential new CPPs will enable more rapid screening for applications such as drug delivery. We have investigated the influence of the composition of training datasets on the ability to classify peptides as cell penetrating using support vector machines (SVMs). We identified 111 known CPPs and 34 known non-penetrating peptides from the literature and commercial vendors and used several approaches to build training data sets for the classifiers. Features were calculated from the datasets using a set of basic biochemical properties combined with features from the literature determined to be relevant in the prediction of CPPs. Our results using different training datasets confirm the importance of a balanced training set with approximately equal number of positive and negative examples. The SVM based classifiers have greater classification accuracy than previously reported methods for the prediction of CPPs, and because they use primary biochemical properties of the peptides as features, these classifiers provide insight into the properties needed for cell-penetration. To confirm our SVM classifications, a subset of peptides classified as either penetrating or non-penetrating was selected for synthesis and experimental validation. Of the synthesized peptides predicted to be CPPs, 100% of these peptides were shown to be penetrating.

## Author Summary

Cell penetrating peptides (CPPs) are peptides that can potentially transport other functional molecules across cellular membranes and therefore serve a role as drug delivery vehicles. The properties of a given peptide that make it cell penetrating are unclear, and the rapid screening of potential CPPs aids researchers by allowing focus on those peptides most likely to be utilized in a therapeutic capacity. This paper shows that basic features representing primary biochemical properties of these peptides can be used to train a classifier that can accurately predict cell penetrating potential of peptides and provide insight into the biochemical properties associated with cell penetration.

## Introduction

Cell penetrating peptides (CPPs), also referred to as “Trojan” peptides, protein transduction domains, or membrane translocation sequences, are typically hydrophobic linear arrangements of 8–24 amino acids able to cross the lipid bi-layer membrane that serves as the cell’s outer barrier and gain access to the interior of the cell and its components [1]. Penetratin, an Antennapedia derived peptide, and the HIV derived Tat peptide were some of the first commonly studied CPPs, and along with transportan peptides (derived from galanin receptor ligand proteins), make up three major families of CPPs. The remainder of CPPs are classified in a fourth, miscellaneous family [1].

Initially, cellular uptake of CPPs was believed to be through endocytosis or protein transporters, but some evidence suggested the mechanism may involve direct transport through the lipid bi-layer of the cell, which takes into account the hydrophobic properties of most of these peptides [2]. The current view is that CPP internalization is accomplished predominantly by endocytosis [2]. Historically, both flow cytometry and fluorescence microscopy have been used to study the uptake of CPPs into cells. Care must be used with these methods to avoid artifacts because traditional methodologies for these techniques can incorrectly show a high concentration of CPPs localizing to the cell nucleus or a higher than actual concentration of CPPs being taken into the cell [2].

Cell penetrating peptides capable of transporting other active molecules inside the cell have the potential to serve as drug delivery peptides. Drug delivery peptides and CPPs allow researchers to probe the mechanisms of peptide transport across a lipid bi-layer membrane and may allow customizable drug therapies for differing types of cells. Although there is some controversy regarding CPPs as drug delivery systems because of their lack of specificity for cell type, the general consensus among researchers is that both general CPPs and cell-specific CPPs will be developed into effective drug delivery systems in the future [3], [4].

A classification system that can determine whether or not a unique peptide sequence can serve as a CPP, and thus possibly be a potential drug delivery peptide, can enable researchers to quickly screen candidate molecules for their potential viability for use in a customizable drug delivery regime.

Much of the previous work in the prediction of CPPs has involved the use of a set of composite features assembled from primary biochemical properties through the use of principal component analysis [5], [6], [7]. These composite features, or *z*-scores, consist of a numerical value and an associated range. To predict cell-penetrating capability of a candidate peptide, the *z*-scores are computed for the peptide, and, if the *z*-scores fall within the range of known CPP *z*-scores, the peptide is classified as cell-penetrating [6], [7]. While this method has a high accuracy (>95% correct prediction of novel CPPs) for generating novel CPPs [6], it performs rather poorly (68% correct prediction) when trying to distinguish known non-penetrating peptides that are closely related to known CPPs [7] and yields little information about exactly which biochemical properties contribute to the difference between these two classes. More recent work examines the use of quantitative structure-activity relationship (QSAR) derived features to predict penetration potential. The training process iteratively removes sequences that are difficult to classify and thus the classification accuracies reported are biased [8]. Further research into this topic is necessary to allow potential drug delivery peptides to be rapidly screened for usefulness.

Using the basic biochemical properties of peptides as features instead of the widely used composite *z*-scores can potentially provide more insight into the differences between the class of CPPs and non-penetrating peptides when coupled with wrapper-based feature selection and classifier training using a machine learning technique such as a support vector machine. Additionally, once trained, these machine learning classifiers can then be used for rapid screening of candidate CPPs prior to their synthesis. This study examines the available information on known CPPs and their non-penetrating analogs in order to compile datasets used for training and testing of support vector machine classifiers using primary features derived from biochemical properties of each peptide and evaluates the accuracy of these classifiers. An experimental validation study was performed to determine the effectiveness of these classifiers using an avian tissue culture system.

## Results/Discussion

The goal of this study was to develop a machine learning approach for rapid screening of potential CPPs. We use features representing primary biochemical properties directly rather than using a transformation such as PCA that combines multiple features into a single composite feature as reported by others [5], [6], [7]. In addition, we have investigated the best approach for constructing training datasets when there is a large disparity in the number of positive and negative examples. Previous research has shown that unbalanced datasets are problematic when constructing classifiers [9]. We first identified known CPPs and known non-penetrating peptides from the literature to serve as positive and negative examples and calculated a number of primary biochemical properties for each of these peptides. We then explored a number of different approaches for addressing the problem of unbalanced datasets and evaluated classification accuracy with the different approaches. A wrapper based feature selection method was utilized to reduce the number of features needed for classification while providing insight into the biochemical properties necessary to distinguish CPPs from non-CPPs. We have used support vector machine classifiers because of their ability to linearly separate classes in a high dimensional feature space. Classifier accuracy on our training sets was assessed using 10-fold cross validation and then each classifier was tested again using the unbalanced test set assembled from the literature. In order to experimentally validate these results, a dataset of 250 peptides was created using a 0^th^ order Markov model based on the predicted chicken proteome [10], and these peptides were classified as either penetrating or non-penetrating by our classifier. Subsets of both predicted penetrating and predicted non-penetrating peptides were selected from these classification results and were synthesized. Experimental validation of cell penetration capability was then determined using fluorescence microscopy and a quantitative uptake study of the peptides was performed.

### Dataset Construction Approaches

Because of the sensitivity of classifiers to unbalanced classes [9], our first challenge was to generate datasets for training and testing. A set of 111 known CPPs were identified from the literature [6], [7], [11]. However, only 34 negative examples could be found and many of these are analogs of known CPPs [6], [7]. Unbalanced datasets present a number of different problems for machine learning methods [9]. When only a comparatively small number of examples are available for one class, the machine learning algorithm will not have sufficient information to learn a function to distinguish the classes. Reporting of classification accuracy is also impacted by unbalanced datasets. For example, if a dataset of 100 peptides contains 80 CPPs and 20 non-CPPs, a classification accuracy of 80% can be obtained by classifying all peptides as positive. Most previous work in CPP prediction has ignored this problem [7], [8].

We designed an experiment to investigate the effect of unbalanced datasets on CPP prediction and to find methods to address the problem to evaluate classifier accuracy with precision. For the CPP prediction problem, there are many more positive examples than negative examples available. Five different approaches were used to generate training datasets for investigating this issue:

1. *Unbalanced:* Composed of 34 known negative examples and 111 known positive examples.
2. *Balanced with random peptides as negative examples*. 111 random peptides were generated using a 0^th^ order Markov chain based on the chicken proteome and combined with 111 known positive examples. All random peptides were assumed to be non-penetrating. This approach is based on the assumption that the probability of randomly generating a CPP sequence is very small.
3. *Balanced with biological peptides as negative examples*. All chicken peptides of length 12–26 AA were downloaded from NCBI and a sample of 111 was drawn without replacement. All were assumed to be non-penetrating. This approach assumes that most biological peptides are non-CPP and the probability of drawing a CPP from this set is extremely low.
4. *Balanced by sampling known negatives*. Random sampling with replacement from the 34 known negatives was used to yield a set of 111 negative examples that was combined with the 111 positive examples.
5. *Balanced by sampling known positives*. Random sampling with replacement from the 111 known positive examples to yield a set of 34 positive examples that was combined with the 34 known negative examples.

### Classifier Performance

The performance of all classifiers on the training data sets is based on 10-fold cross validation. The confusion matrices for classifiers trained using datasets based on approaches 1–4 are shown in Table 1 and the classifier statistics are shown in Tables 2 and 3. The classifier trained on the unbalanced dataset (111 positive examples and 34 negative examples) has a classification accuracy of only 75.86% compared to the naïve approach of classifying all examples as positive which would result in a classification accuracy of 76.55%. The results for this dataset in Table 1 show that the resulting classifier predicts almost all examples to be positive. This highlights the problems encountered when using an unbalanced dataset. The classifier cannot distinguish positive and negative examples because the dataset contains so many more positive examples than negative examples and because many of the negative examples are analogs of the positives.

**Table 1:** Confusion matrices for datasets generated using different approaches.

	Non-CPP	CPP	←Classified as
Dataset 1 – Unbalanced.			
(total examples 145)	0	34	Non-CPP
	1	110	CPP

Dataset 2 – Balanced with random peptides as negatives.			
10-fold cross validation with training data (total examples 222)	109	2	Non-CPP
	7	104	CPP
Tested on unbalanced data (total examples 145)	12	22	Non-CPP
	6	105	CPP

Dataset 3 – Balanced with biological peptides as negatives.			
10-fold cross validation with training data (total examples 222)	108	3	Non-CPP
	10	101	CPP
Tested on unbalanced data(total examples 145)	10	24	Non-CPP
	6	105	CPP

Dataset 4 – Balanced by sampling known negatives.			
10-fold cross validation with training data (total examples 222)	96	15	Non-CPP
	10	101	CPP
Tested on unbalanced data (total examples 145)	29	5	Non-CPP
	7	104	CPP

**Table 2:** Classifier performance with different training regimes - Performance from ten-fold cross validation with training data sets.

	Unbalanced	Balanced with random negatives	Balanced with biological negatives	Balanced by sampling from known negatives	Balanced by sampling from known positives*
Accuracy	75.86%	95.94%	94.14%	88.73%	78.82%
True Positive Rate	0.759	0.959	0.941	0.887	0.7883
False Positive Rate	0.768	0.041	0.059	0.113	0.2117
ROC	0.495	0.959	0.941	0.887	0.7883

*- These values represent the averages for 10 datasets.

**Table 3:** Classifier performance of each classifier with original dataset.

	Unbalanced	Balanced with random negatives	Balanced with biological negatives	Balanced by sampling from known negatives
Accuracy	75.86%	80.69%	79.31%	91.70%
True Positive Rate	0.759	0.807	0.793	0.917
False Positive Rate	0.768	0.508	0.553	0.127
ROC	0.495	0.649	0.620	0.895

The classifiers trained using both the dataset balanced with random peptides for negatives (approach 2) and with biological peptides for negatives (approach 3) had classification accuracies of 95.95% and 94.14% respectively, indicating that both classifiers exhibit a high degree of accuracy in discriminating between known cell-penetrating peptides and randomly generated or biological peptides assumed to be negative. The confusion tables for these classifiers on the training data sets (Table 1) show that most of the mistakes are false negatives (CPPs incorrectly classified as non-CPPs). The weakness of these training approaches is that some of the assumed negative examples may in fact be cell penetrating and known non-cell penetrating analogs of CPPs were not used as negative examples. When we used these trained classifiers to evaluate the known non-penetrating cell penetrating analog peptides (our unbalanced test data set) these classifiers obtained accuracies of 80.69% and 79.31% respectively. For both classifiers, approximately one third of the known non-penetrating peptides are classified as cell-penetrating. Most of the mistakes made by these two classifiers on the test data seem to be false positives, that is classifying a peptide with no cell penetrating potential as a CPP, and this classification of known non-penetrating cell penetrating analogs demonstrates that while these classifiers are very accurate distinguishing the features strongly predictive of cell penetrating potential from the vast majority of non-penetrating peptides, the features used for classification do not serve to distinguish between peptides more similar to CPPs that do not penetrate and those peptides that can act as CPPs.

The classifier trained on the data set constructed using approach 4 (random sampling with replacement from the known negative examples) has a classification accuracy of 88.74% on the training data set when evaluated with 10-fold cross validation. When compared to the classification accuracy of the dataset generated using the unbalanced dataset, these results show that it is possible to classify a set of CPPs and a set of known non-penetrating peptides using our SVM based method when care is used to construct balanced datasets. Tables 2 and 3 show that 60% of the errors are false positives (non-CPPs incorrectly classified as CPPs). When we evaluated the unbalanced test set on this classifier, an accuracy of 91.72% was obtained. The classifiers trained on the smaller datasets using approach 5 have an average classification accuracy of 78.82% using 10-fold cross validation.

Approach 2 using randomly selected biological peptides as the negative examples gives the best 10-fold cross validation accuracy while approach 4 with random selection from the negative examples gives the best accuracy for the unbalanced training set. This suggests use of a two step process for screening. In the first step, a classifier trained with random biological peptides as the negative examples would be used for preliminary bulk screening. As a second step, peptides predicted to be CPP in step 1 would be screened by a classifier trained using approach 4 that is more accurate in distinguishing non-penetrating analogs from CPPs. Approach 4 also provides more insight into the rational design of novel CPP analogs as the negative examples used in this approach are generally constructed by the modification of a known CPP sequence.

In Hällbrink et al. (2005), the authors describe a method of CPP prediction based on scoring a candidate peptide according to *z*-score descriptors, features compiled through PCA, and report an 84.05% accuracy in the prediction of 53 CPPs and 16 non-functional CPP analogs [6]. A follow-up to this study, utilizing both more known CPPs (65) and more non-functional CPP analogs (20), reports a 68% prediction efficiency using the same *z*-score descriptor based prediction method [7]. More recently, these *z*-score descriptors were utilized alongside quantitative structure-activity relationship features in an artificial neural network (ANN) to predict cell penetrating potential for a set of 101 peptides (77 CPPs, 24 non-penetrating CPP analogs) and report a classification accuracy of 83% for the general ANN model constructed [8]. However, it should be noted that the data set utilized is composed of unbalanced classes, and an accuracy of 76.24% can be achieved by classifying every peptide encountered as a CPP. A comparison of these previously published prediction methods and our approach is presented in Table 4. The models constructed using our approaches and their high classification accuracies indicate that using the primary biochemical properties of a peptide as features instead of synthesized feature values compiled using PCA allows for a more informative analysis of which properties determine whether a given peptide is cell-penetrating. In contrast to PCA approaches where complex features are constructed in both the feature selection step and again for classifier construction, our consistent use of the SVM for wrapper-based feature selection and for classifier construction, allows predictive models to be constructed to provide for more rapid and elucidative screening of cell-penetrating potential than previous predictive methods.

**Table 4:** Comparison of SVM based CPP classifiers to previously published methods.

	Hällbrink-2005 [6]	Hansen-2008 [7]	Dobchev-2010 [8]	Unbalanced	Distribution-based	Biologically-based	Balanced by sampling Non-CPPs
Overall Accuracy	77.27%	67.44%	83.16%	75.86%	80.69%	79.31%	91.72%
CPP Accuracy	88.46%	80.30%	92.21%	99.10%	94.59%	94.59%	93.69%
Non-CPP Accuracy	35.71%	25.00%	54.17%	0.00%	35.29%	29.41%	85.29%

For each classifier constructed, feature selection was conducted using a scatter search approach through feature space [12] where the “wrapped” classifier was the same type of SVM used for classifier construction. The classifier is a sequential minimal optimization SVM [13] using the Pearson Universal Kernel [14]. Table 5 lists the features selected for datasets 1–4 above. Because the number of training/testing samples for dataset 5 was so small, we generated ten different datasets using this approach. The features selected from these ten datasets are listed in Table 6. The features selected for the datasets constructed using approaches 1–5 contain a number of properties previously shown to aid in the prediction of CPPs. These include net charge, positive charge, negative charge, the net donated hydrogen bonds, and the water-octanol partition coefficient. The low number of features selected for the datasets constructed using approach 5 indicates over-fitting of these small datasets by the classification algorithm. Therefore our detailed examination of features selected focused on datasets generated using approaches 1–4. The primary amino acid composition features, the number of a given amino acid and the percent a given amino acid contributes to the whole peptide sequence, indicates no predictive function arising from the non-polar amino acids leucine and isoleucine, the polar amino acid glutamine, and the negatively charged amino acid glutamate. At least one of the amino acid composition features was selected for the remaining amino acids, with the most notable of these being the positively charged amino acids lysine, arginine, and histidine, and the negatively charged amino acid aspartate. In addition, the group of aromatic amino acids were selected to a notable degree, and the presence of some aromatic amino acids in the peptide sequence has been previously reported to be required for cell-penetrating potential [15].

**Table 5:** Features selected for datasets generated using approaches 1–4.

Dataset 1(Balanced with random negative examples)	Dataset 2(Balanced with biological peptides assumed to be negative)	Dataset 3(Unbalanced dataset)	Dataset 4(Balanced by random sampling of known negatives with replacement)
Net Charge	Net Charge	Net Charge	Negative Charge
Positive Charge	Isoelectric Point	Positive Charge	Isoelectric Point
Number of serines (S)	Molecular Weight	Number of alanines (A)	Number of glycines (G)
Number of aspartates (D)	Hydropathicity	Number of arginines (R)	Number of alanines (A)
Percent valine (V)	Number of valines (V)	Percent arginines (R)	Number of tryptophans (W)
Percent proline (P)	Number of lysines (K)	Net Donated Hydrogen Bonds	Number of asparagines (N)
Percent phenylalanine (F)	Number of arginines (R)		Number of lysines (K)
Percent threonine (T)	Percent glycine (G)		Number of histidines (H)
Percent asparagine (N)	Percent methionine (M)		Number of aspartates (D)
Percent tyrosine (Y)	Percent tyrosine (Y)		Percent phenylalanine (F)
Percent cysteine (C)	Percent cysteine (C)		Percent tryptophan (W)
Percent arginine (R)	Percent aspartate (D)		Percent arginine (R)
Percent histidine (H)	Percent negative		Percent histidine (H)
Percent aspartate (D)	Water Octanol Partition Coefficient		Percent Hydrophobic
Percent negative	Net Donated Hydrogen Bonds		Percent negative
Steric Bulk	Percent Helix		Hydrophobicity
Net Donated Hydrogen Bonds	Percent Coil		Water Octanol Partition Coefficient
Percent Helix			
Percent Coil			

**Table 6:** Features selected for ten datasets generated using approach 5.

Dataset 1	Dataset 2	Dataset 3	Dataset 4	Dataset 5	Dataset 6	Dataset 7	Dataset 8	Dataset 9	Dataset 10
Number (V)	Length	Number (R)	Net Charge	Net Charge	Percent (T)	Net Charge	Positive Charge	Number (W)	Positive Charge
Percent (R)	Net Charge	Percent (W)	Negative Charge	Percent (I)	Percent (Y)	Positive Charge	Number (G)	Number (T)	Percent (I)
	Number (V)	Percent positive	Number (I)	Hydrophobicity	Net Donated Hydrogen Bonds	Percent (I)	Number (S)	Number (R)	Amphipacity
	Number (C)	Amphipacity	Number (H)	Net Donated Hydrogen Bonds	Percent Sheet	Percent (W)	Percent (F)	Percent (S)	
	Percent (H)	Percent Helix	Percent (F)			Percent Hydrophobic	Percent (R)	Percent (T)	
	Net Donated Hydrogen Bonds		Net Donated Hydrogen Bonds				Percent (H)		
							Amphipacity		

Balanced subsets of CPPs sampled with replacement combined with known-CPP analogs.

### Validation Study

To experimentally validate our feature selection methodology and classifiers, 250 random peptides were generated using a 0^th^ order Markov model based on the chicken predicted proteome and were classified as penetrating or non-penetrating using the classifier trained on the dataset constructed using random peptides as negative examples. From these classifications, four peptides predicted to be cell-penetrating and two peptides predicted to be non-penetrating were selected for synthesis and FITC-labeling along with three known cell penetrating peptides used for positive controls, three peptides consisting respectively of only polar amino acids, only non-polar amino acids, and only of mixed polar and non-polar amino acids to serve as negative controls. In addition, a known non-penetrating peptide (TP13, a transportan analog [15]) was selected for synthesis to serve as a minor validation for our set of known non-penetrating peptides.

#### Cellular internalization microscopy array of FITC-labeled peptides

**Figure 1:**
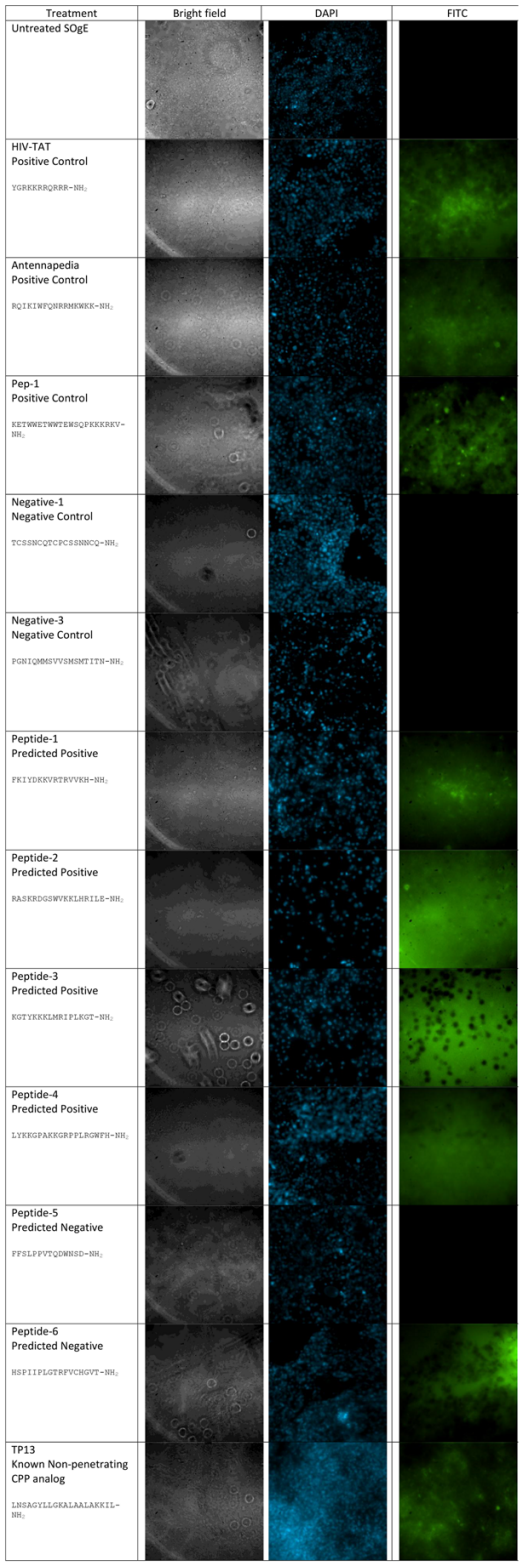
Cellular internalization microscopy array of FITC-labeled peptides.

The uptake of synthesized FITC-labeled peptides was examined using an avian system to validate both our wrapper based feature selection methodology and SVM-based approach to predicting CPPs. The results of our fluorescence microscopy analysis are shown in Figure 1. All peptides predicted to be cell-penetrating (Peptide-1 through Peptide-4) by our classifier were confirmed to be cell-penetrating. Of our two negative predictions, Peptide-5 was confirmed to be a non-penetrating peptide while Peptide-6 was shown to traverse cellular membranes. TP13, a CPP analog previously shown to be non-penetrating in Bowes’ melanoma cells is clearly cell-penetrating peptide in our avian model.

#### Uptake quantification of FITC-labeled peptides

**Figure 2:**
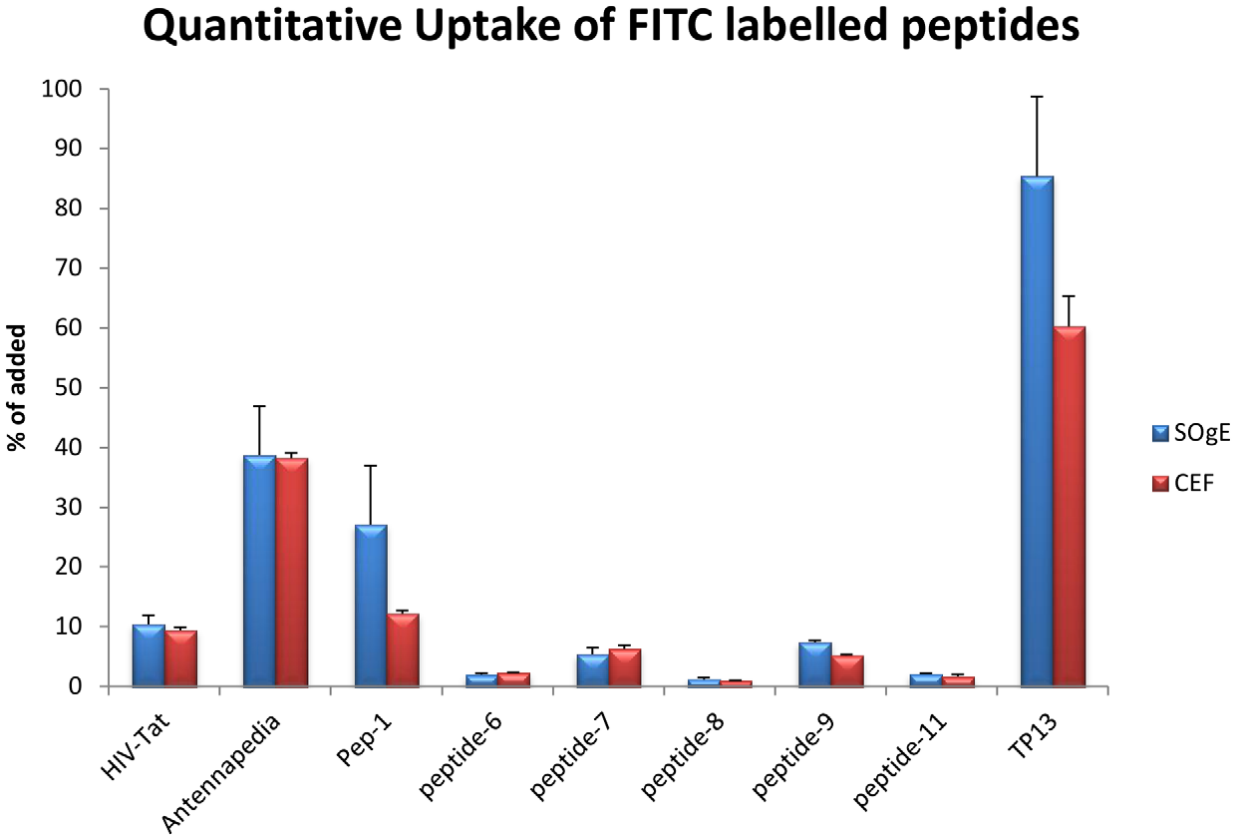
Quantitative uptake analysis.

To evaluate the relative uptake of our synthesized peptides and to provide a secondary confirmation of the fluorescence microscopy results, a quantitative uptake study was conducted using both quail SOgE cells and chicken embryonic fibroblasts. The results of the quantitative uptake study for those peptides shown to be penetrating (p≤0.05) are shown in Figure 2. Peptides 1–4 were shown to be CPPs, while Peptide-5 was correctly predicted to be non-penetrating. Peptide-6, which was predicted to be non-penetrating, was shown to traverse the membranes of both CEF and SOgE cells. TP13, previously shown to be non-penetrating in melanoma cells, is again shown to have penetrated both CEF and SOgE cells to a high degree relative to both our positive controls and our predicted cell-penetrating peptides.

TP13 was chosen as a non-penetrating CPP analog based on its non-CPP classification in a study examining the effects of deletion on a known CPP, transportan (TP) [15]. TP13 was created by a deletion from the N-terminus and middle of the TP molecule and these deletions abolished the internalization of TP13 into Bowes’ melanoma cells. All transportan-derived peptides that internalized during the original TP analog study contained tyrosine and 3 positive charges in their sequences, while those peptides without tyrosine or one positive charge in the C-terminal portion of the peptide did not internalize [15]. TP13 contains tyrosine and 3 positive charges, meeting the criteria outlined by the original study for penetration and both our fluorescent microscopy data and quantitative fluorescent uptake data indicates that it does penetrate both SOgE cells and CEF cells.

Peptide-6 (HSPIIPLGTRFVCHGVT) was predicted to be a non-CPP by our classifier, but was shown to internalize into both SOgE and CEF cells experimentally both by fluorescence microscopy and the quantitative uptake studies. This peptide contains 3 positively charged amino acids along with phenylalanine. The Sommets, *et al*. study examining TP and its derivatives states that all their peptides with 3 positive charges and tyrosine internalized, and as phenylalanine only lacks the hydroxyl group of the tyrosine molecule, this could contribute to the internalization of Peptide-6. The positive examples in our training data contain predominantly arginine and lysine as positive residues, while this peptide contains two histidine residues.

Our research shows that using the primary biochemical properties of peptides as features instead of composite features determined through the use of PCA can provide both more informative features and higher classification accuracies when using support vector machines for the classification of a given peptide as cell-penetrating. The lack of a comprehensive and coherent database of cell-penetrating peptide data for bioinformatics analysis has been noted previously [7], and the majority of CPP studies have been conducted using a variety of different cell lines and detection techniques, making it difficult to unify these results. Our results showing that a previously reported non-penetrating analog of transportan is a CPP in our avian system confirms the need for a large dataset of biologically confirmed positive and negative examples from a single biological system using a single detection methodology. Until such a resource is available, the predictive capability of classifiers is difficult to assess. Our results also show that there may be classes of peptides that act as CPPs in a variety of cells and others that are more specialized. Therefore, peptides designed to target delivery to specific cells and tissues of interest should be screened using a variety of cell lines. Additionally, our results indicate there may be positional preference for certain types of amino acids such as positive charges and aromatic. Further research should examine the effects of these positional effects. Also, there are several classes of CPPs which may not utilize the same internalization mechanism, and future research could focus on developing classifiers for each of these individual CPP classes. A problem that arises from the current set of known CPPs is the small overall size may not yield many examples to build distinct classifiers for different internalization mechanisms. Certain CPPs may be more capable of delivering certain classes of cargos across cellular membranes, and may internalize to different cellular locations once internalized, and future research could focus predicting which CPPs are best suited to transport of particular cargos and predicting where within a cell they may internalize. Additionally, different CPPs can penetrate certain types of cells, and in the future, information about the various membrane components (lipid and membrane protein composition/concentration) of cells of interest could be incorporated into classifiers. The primary problem is the small set of known CPPs and non-penetrating CPP analogs is assembled from a number of different experimental techniques (different detection methodologies, different cell types, etc), and there is a great need for the creation of a dataset of CPPs evaluated in a number of different cell types of interest and evaluated under the same types of experimental conditions for cell penetration, cargo carrying capability, and internal localization once penetration has occurred.

## Materials and Methods

### Data Set Compilation Strategy

A database of cell-penetrating peptides was constructed from the literature and from commercial vendor product lines [6], [7], [11]. A total of 111 cell-penetrating peptide (CPP) sequences were identified and used to create a database of positive examples (Table 7) [6], [7], [11]. The average amino acid lengths of these CPPs ranged from 12 to 26. Because very few peptides have been experimentally validated to be non-penetrating, it was more challenging to construct a database of negative examples. Five different strategies were used. Because our experimental system is avian, we have used the composition of the chicken proteome as the basis for two of our datasets. Previous research has demonstrated the importance of using a balanced training sets where there are approximately equal numbers of positive and negative examples [9].

**Table 7:** Known cell-penetrating peptides from the literature and commercial vendors.

Cell-penetrating peptide	Reference
AAVALLPAVLLALLAKNNLKDCGLF	[11]
AAVALLPAVLLALLAKNNLKECGLY	[11]
AAVALLPAVLLALLAPVQRKQKLMP	[11]
AAVALLPAVLLALLAVTDQLGEDFFAVDLEAFLQEFGLLPEKE	[11]
AAVLLPVLLAAP	[7], [11]
AGYLLGKINLKALAALAKKIL	[6], [7]
AGYLLGKLKALAALAKKIL	[7]
AHALCLTERQIKIWFQNRRMKWKKEN	[7]
AHALCPPERQIKIWFQNRRMKWKKEN	[7]
ALWKTLLKKVLKA	[6]
AYALCLTERQIKIWFANRRMKWKKEN	[7]
CGPGSDDEAAADAQHAAPPKKKRKVGY	[7]
CNGRC	[11]
CNGRCG	[11]
CNGRCGGKKLKLLKLL	[11]
CNGRCGGKLAKLAKLAKLAK	[11]
CNGRCGGLVTT	[11]
GAARVTSWLGRQLRIAGKRLEGRSK	[6]
GALFLGFLGAAGSTMGAWSQPKSKRKV	[11]
GGRQIKIWFQNRRMKWKK	[6]
GIGKFLHSAKKWGKAFVGQIMNC	[11]
GLAFLGFLGAAGSTMGAWSQPKSKRKV	[7]
GRKKRRQ	[6]
GRKKRRQRRPPQC	[7]
GRKKRRQRRRC	[6], [7]
GRKKRRQRRRPPC	[6], [7]
GRKKRRQRRRPQ	[6], [7]
GRQLRIAGKRLEGRSK	[6]
GWTLNPAGYLLGKINLKALAALAKKIL	[6], [7]
GWTLNPPGYLLGKINLKALAALAKKIL	[6], [7]
GWTLNSAGYLLGKINLKALAALAKKIL	[6], [7], [11]
GWTLNSAGYLLGKINLKALAALAKKLL	[6], [7]
GWTLNSAGYLLGKLKALAALAKKIL	[6], [7]
GWTLNSKINLKALAALAKKIL	[7]
INLKALAALAKKIL	[11]
IWFQNRRMKWKK	[7]
KALAALLKKWAKLLAALK	[7]
KALAKALAKLWKALAKAA	[6], [7]
KALKKLLAKWAAAKALL	[6], [7]
KCRKKKRRQRRRKKLSECLKRIGDELDS	[6]
KCRKKKRRQRRRKKPVVHLTLRQAGDDFSR	[6]
KETWWETWWTEWSQPKKKRKV	[11]
KETWWETWWTEWSQPKKRKV	[7]
KFHTFPQTAIGVGAP	[7]
KITLKLAIKAWKLALKAA	[6], [7]
KIWFQNRRMKWKK	[7]
KLAAALLKKWKKLAAALL	[6], [7]
KLALKALKALKAALKLA	[6], [7]
KLALKLALKALKAALK	[6], [7]
KLALKLALKALQAALQLA	[7]
KLALKLALKAWKAALKLA	[6], [7]
KLALQLALQALQAALQLA	[7]
KMTRAQRRAAARRNRWTAR	[6]
KRPAATKKAGQAKKKKL	[6]
LGTYTQDFNKFHTFPQTAIGVGAP	[7]
LIRLWSHLIHIWFQNRRLKWKKK	[7]
LKTLATALTKLAKTLTTL	[7]
LKTLTETLKELTKTLTEL	[7]
LLGDFFRKSKEKIGKEFKRIVQRIKDFLRNLVPRTESC	[7]
LLIILRARIRKQAHAHSK	[6]
LLIILRRPIRKQAHAHSK	[6]
LLIILRRRIRKQAHAHSA	[6]
LLIILRRRIRKQAHAHSK	[6], [7]
LNSAGYLLGKINLKALAALAKKIL	[6], [7]
LNSAGYLLGKLKALAALAKIL	[7]
MANLGYWLLALFVTMWTDVGLCKKRPKP	[7]
MDAQTRRRERRAEKQAQWKAAN	[6], [11]
MGLGLHLLVLAAALQGAKKKRKV	[6]
MPKKKPTPIQLNP	[11]
MVKSKIGSWILVLFVAMWSDVGLCKKRPKP	[7]
MVTVLFRRLRIRRACGPPRVRV	[7]
NAKTRRHERRRKLAIER	[6], [11]
PKKKRKV	[11]
PKKKRKVALWKTLLKKVLKA	[6]
PMLKE	[7]
QLALQLALQALQAALQLA	[7]
RGGRLSSYSRRRFSTSTGR	[7]
RGGRLSYSRRRFSTSTGR	[6]
RGGRLSYSRRRFSTSTGRA	[11]
RKKRRQRRR	[6], [7]
RKSSKPIMEKRRRAR	[6]
RQARRNRRRALWKTLLKKVLKA	[6]
RQGAARVTSWLGRQLRIAGKRLEGR	[6]
RQGAARVTSWLGRQLRIAGKRLEGRSK	[6]
RQIKIWFPNRRMKWKK	[6], [7]
RQIKIWFQNMRRKWKK	[7]
RQIKIWFQNRRMKWKK	[6], [7], [11]
RQIKIWFQNRRMKWKKLRKKKKKH	[6]
RQIRIWFQNRRMRWRR	[7], [11]
RQPKIWFPNRRMPWKK	[7]
RRLSSYSSRRRF	[7]
RRMKWKK	[7]
RRRRRRRRR	[6], [7], [11]
RRWRRWWRRWWRRWRR	[7]
RVIRVWFQNKRCKDKK	[6], [7]
RVTSWLGRQLRIAGKRLEGRSK	[6]
SWLGRQLRIAGKRLEGRSK	[6]
TAKTRYKARRAELIAERR	[6], [11]
TRQARRNRRRWRERQR	[7]
TRRNKRNRIQEQLNRK	[6], [7], [11]
TRSSRAGLQFPVGRVHRLLRK	[11]
TRSSRAGLQWPVGRVHRLLRKGGC	[11]
VPALR	[7]
VPMLK	[7]
VPTLK	[7]
VQAILRRNWNQYKIQ	[6]
VRLPPPVRLPPPVRLPPP	[7]
WFQNRRMKWKK	[7]
YGRKKRRQRRR	[11]
YGRKKRRQRRRGTSSSSDELSWIIELLEK	[6]
YGRKKRRQRRRSVYDFFVWL	[6]

#### Balanced with random peptides

The set of 111 know CPPs was balanced with a set of 111 peptides constructed using a 0^th^ order Markov chain derived from the IPI chicken proteome (ipi.CHICK.v3.56 [10]) . The peptide lengths were uniformly distributed in the range 12–26. We assume that there is a very low probability that randomly generated peptides would be cell penetrating.

#### Balanced with biological peptides

The set of 111 know CPPs was balanced with randomly selected biological peptides. A set of 411 chicken peptides from NCBI with lengths in the range 12–26 was downloaded. Subsets of 111 peptides were selected randomly without replacement to provide multiple balanced datasets. This dataset provides a set of positive examples of known CPPs and assumed negative examples of biological peptides of the same relative molecular size. We assume that most naturally peptides are not cell penetrating.

#### Unbalanced using only known positives

A set of 34 known non-penetrating cell penetrating peptide analogs and peptide hormones previously used as negative examples was constructed from a search of the literature and are listed in Table 8 [6], [7]. This dataset provides a set of known cell-penetrating positive examples and a set of non-penetrating peptides that have been experimentally shown not to traverse cellular membranes.

**Table 8:** Known non-penetrating cell-penetrating peptide analogs and peptide hormones.

Non-cell penetrating peptide	Reference
AGCKNFFWKTFTSC	[6]
AHALCLTERQIKSNRRMKWKKEN	[7]
CYFQNCPRG	[6]
DFDMLRCMLGRVYRPCWQV	[6]
EILLPNNYNAYESYKYPGMFIALSK	[6]
FITKALGISYGRKKRRQC	[7]
FVPIFTHSELQKIREKERNKGQ	[6]
GRKKRRQPPQC	[7]
GWTLNSAGYLLGKFLPLILRKIVTAL	[6], [7]
GWTLNSAGYLLGKINLKAPAALAKKIL	[6], [7]
GWTLNSAGYLLGPHAI	[6]
GWTNLSAGYLLGPPPGFSPFR	[6]
HDEFERHAEGTFTSDVSSYLEGQAAKEFIAWLVKGR	[6]
IAARIKLRSRQHIKLRHL	[7]
ILRRRIRKQAHAHSK	[7]
KIWFQNRRMK	[7]
KKKQYTSIHHGVVEVD	[6]
KKLSECLKRIGDELDS	[6]
KLALKALKAALKLA	[6], [7]
KLALKLALKALKAA	[7]
LLGKINLKALAALAKKIL	[7]
LLKTTALLKTTALLKTTA	[6],
LLKTTELLKTTELLKTTE	[6], [7]
LNSAGYLLGKALAALAKKIL	[6], [7]
LNSAGYLLGKLKALAALAK	[6], [7]
LRKKKKKH	[6]
PVVHLTLRQAGDDFSR	[6]
QNLGNQWAVGHLM	[6]
RPPGFSPFR	[6]
RQIKIFFQNRRMKFKK	[6], [7]
RQIKIWFQNRRM	[7]
RQIKIWFQNRRMKWK	[7]
TERQIKIWFQNRRMK	[7]
WSYGLRPG	[6]

#### Balanced by sampling known negatives

In order to produce a balanced dataset of both known non-penetrating peptides and known CPPs a set consisting of all 111 known cell penetrating peptides and 111 known non-penetrating cell penetrating analogs was constructed by selecting with replacement from the set of 34 known non-penetrating analogs .

#### Balanced by sampling known positives

Subsets of the known CPPs of size 34 were selected with replacement and combined with the 34 known non-penetrating cell penetrating analogs to create ten balanced subsets.

### Feature Construction and Normalization

For each dataset, we generate a set of basic biochemical properties of each peptide (e.g. mass, size, charge, secondary structure, etc) and other features previously shown to be useful in the prediction of CPPs (e.g. steric bulk and net donated hydrogen bonds) [7]. The full list of the initial 61 features is shown in Table 9. We use these features directly in our machine learning algorithm rather than using composite features such as features derived by principle component analysis (citation). We feel this approach will be more informative in the rationale design of CPPs cell penetrating peptides. Because the data values for each feature within a dataset vary greatly, NV normalization was used to scale the numeric range of all features in the range [0, 1] [16].

**Table 9:** A list of initial features used for classifier construction.

Feature	Reference
Length of peptide	[22]
Net charge of peptide	[22]
Positive charge	[22]
Negative charge	[22]
Isoelectric point (pI)	[22]
Molecular weight	[22]
Hydropathicity	[23]
Number of Each Amino Acid (20 features)	[22]
Percent composition of each amino acid (20 features)	[22]
Percent polar amino acids	[22]
Percent positive amino acids	[22]
Percent negative amino acids	[22]
Percent hydrophobic amino acids	[22]
Hydrophobicity	[23]
Lipophilicity	[24]
Amphiphilicity	[25]
Water-Octanol Partition Coefficient	[23]
Steric Bulk	[23]
Side chain bulk	[7]
Net donated hydrogen bonds	[7]
Percent α helix	[26]
Percent random coil	[26]
Percent β sheet	[26]

### Machine Learning Software

The WEKA Machine Learning Toolkit Version 3.6.1, a freely available software package containing a number of machine learning algorithms for data mining, was used for feature selection, classifier construction, and classifier evaluation [17].

### Feature Selection

We conducted feature selection to reduce the dimensionality of the feature vectors. Empirical evaluation of a number of different feature selection methods was conducted and the best performance was obtained using a wrapper-based method. The wrapper-based method uses a parallel scatter search algorithm [12] to evaluate feature subsets based on classifier performance. Scatter search is an evolutionary algorithm, but unlike other evolutionary algorithms (e.g. genetic algorithms), the search for a local optimum is guided through the use of a reference set that acts to intensify and diversify the resulting features [12]. Local searches of features generated from the reference set are conducted, and informative and diverse features from these local searches are used to update the reference set until a terminating condition is met [12].

### Classifier Construction

Our classifier is a support vector machine (SVM) trained via a sequential minimal optimization (SMO) algorithm used in conjunction with the Pearson VII universal kernel [13], [14]. SVMs are supervised learning classifiers generally used for solving two class problems, and in their simplest form can be thought of as a classifier separating two classes mapped onto a 2-dimensional plane by generating a line through the plane that optimizes the distribution of each class on either side of the line [13]. The SMO algorithm is a modification to the original SVM learning algorithms that replaces a numerical quadratic programming step with an analytical quadratic programming step, allowing the algorithm to spend a greater portion of time on the decision function instead of the quadratic programming step. This greatly increases the speed of the SVM for classification and allows scaling for large datasets [13]. We chose to utilize SMO-based SVM classifiers because of their speed and performance for our two class problem of determining if given peptide is cell-penetrating or non-penetrating. A kernel function used in conjunction with an SVM allows the classifier to examine non-linear relationships between features by mapping the initial non-linear features into a highly dimensional space where the solution can be represented by a linear classification [14]. We chose the Pearson VII universal kernel (PUK) for our SMO-based SVM because PUK has been shown to provide either equal or better mapping than traditional SVM kernels, while serving as a robust and generic alternative to other kernel functions [14]. Accuracy for all classifiers was evaluated using 10-fold cross-validation.

### Peptide Synthesis

A 0^th^ order Markov chain based on the amino acid frequency of the IPI Chicken Proteome (ipi.CHICK.v3.56) [10] was used to generate 250 peptides. The classifier trained on our biologically based random peptide dataset was then used to classify each of these peptides. From these classification results, four peptides predicted to be cell penetrating and two peptides predicted to be non-cell penetrating were selected for synthesis and experimental validation. In addition, three peptides known to be cell-penetrating (HIV-Tat [18], Antennapedia [19], and Pep-1 [20]) were chosen to be positive experimental controls. Three other peptides, one of all polar amino acids, one of all non-polar amino acids, and one of a mix of polar and non-polar amino acids, were chosen as negative experimental controls because their lack of charged and aromatic R-groups make it unlikely they would cross a cellular membrane. One peptide (TP13 [7], [15]) was randomly selected for synthesis from the list of known non-penetrating cell penetrating peptide analogs. All peptides selected for synthesis are shown in Table 10. Peptides were synthesized (>95% purity) and N-terminally labeled with FITC, a fluorescent tag, by Biomatik. During the peptide synthesis, one of our chosen negative controls, negative-2 (GLALLGIAVAILVVL-NH_2_) was unable to be synthesized to our desired purity levels due to insolubility issues and is not considered further. The lyophilized peptides were reconstituted using 1 mL of 4∶1 dd H_2_O sterile filtered 0.45 µm and acetonitrile (EMD OmniSolv).

**Table 10:** Peptides synthsized for experimental validation of classifier.

Name	Role	Sequence (N to C)
HIV-TAT [18]	Control(+)	YGRKKRRQRRR-NH_2_
Antennapedia [19]	Control(+)	RQIKIWFQNRRMKWKK-NH_2_
Pep-1 [20]	Control(+)	KETWWETWWTEWSQPKKKRKV-NH_2_
negative-1	Control(-)	TCSSNCQTCPCSSNNCQ-NH_2_
negative-2*	Control(-)	GLALLGIAVAILVVL-NH_2_
negative-3	Control(-)	PGNIQMMSVVSMSMTITN-NH_2_
peptide-1	Predicted CPP	FKIYDKKVRTRVVKH-NH_2_
peptide-2	Predicted CPP	RASKRDGSWVKKLHRILE-NH_2_
peptide-3	Predicted CPP	KGTYKKKLMRIPLKGT-NH_2_
peptide-4	Predicted CPP	LYKKGPAKKGRPPLRGWFH-NH_2_
peptide-5	Predicted Non-CPP	FFSLPPVTQDWNSD-NH_2_
peptide-6	Predicted Non-CPP	HSPIIPLGTRFVCHGVT-NH_2_
TP13 [7], [15]	Known Non-CPP-CPP Analog	LNSAGYLLGKALAALAKKIL-NH_2_

*negative-2 was unable to be synthesized to desired purity levels due to insolubility issues.

### Tissue Culture

Two avian cell lines, Quail SOgE muscle cells [21] and a primary culture of Chicken embryonic fibroblasts (CEF), were grown in tissue culture flasks in Dulbecco’s minimal essential medium containing 10% fetal bovine serum with penicillin (200 IU/mL), streptomycin (200 µg/mL), amphotericin B (0.5 µg/mL) (MP Biomedicals), and non-essential amino acids at 37°C in a 5% CO_2_ atmosphere .

### Quantitative Uptake Analysis

Approximately 100,000 cells per well (both CEFs and SOgEs) were plated onto 12-well tissue culture plates approximately 2 days prior to the experiment and allowed to reach confluency. The cells were changed to serum free media and incubated for 60 minutes prior to experimentation. The cells were then washed with two 1 mL washes of PBS, after which they were exposed to 300 µL of 10 µM peptide in serum free media for 30 minutes, with three replicates per peptide per cell line. The cells were then washed with two 1 mL washes of PBS, and lightly trypsinated to remove any external peptides that may have been attached to the cellular membrane and facilitate the detachment of cells from the tissue culture flask. Centrifugation of the cells was performed at 250 x G for 4 min, and the supernatant aspirated off. Cells were then lysed with 250 µL of 0.1% Triton-X in PBS at 4° C for 10 minutes. A 100 µL aliquot of the cell lysate and a 100 µL aliquot of the 10 µM peptide in serum free media were pipetted onto a 96-well plate. Fluorescence was measured on a Dynex Fluorolite 1000 plate reader at 485/530 nm. The samples were compared to the fluorescence of the added amount of peptide and *t*-tests (p≤0.05) were performed for each experimental sample against an untreated control.

### Cellular Internalization Microscopy Array of FITC-Labeled Peptides

The SOgE cells were seeded onto glass tissue microscopy slides (approximately 50,000 cells/well), and allowed two days to reach confluency. The cells were changed to serum free media and incubated for 60 minutes prior to experimentation. The cells were then washed with two 1 mL washes of PBS, after which they were exposed to 300 µL of 10 µM peptide in serum free media for 30 minutes. The cells were then washed with two 1 mL washes of PBS, and then fixed using UltraCruz™Mounting Medium (Santa Cruz Biotechnology) containing a DAPI nuclear stain. The fluorescence was examined using a Nikon Eclipse TE2000-U Inverted Research Microscope with the MetaMorph microscopy imaging software.

## References

[1] Kilk K (2004). Cell-penetrating peptides and bioactive cargoes.. Strategies and mechanisms.

[2] Richard JP, Melikov K, Vives E, Ramos C, Verbeure B (2003). Cell-penetrating peptides. A reevaluation of the mechanism of cellular uptake.. J Biol Chem.

[3] Schwartz JJ, Zhang S (2000). Peptide-mediated cellular delivery.. Curr Opin Mol Ther.

[4] Vives E (2005). Present and future of cell-penetrating peptide mediated delivery systems: “is the Trojan horse too wild to go only to Troy?”.. J Control Release.

[5] Sandberg M, Eriksson L, Jonsson J, Sjostrom M, Wold S (1998). New chemical descriptors relevant for the design of biologically active peptides. A multivariate characterization of 87 amino acids.. J Med Chem.

[6] Hallbrink M, Kilk K, Elmquist A, Lundberg P, Lindgren M (2005). Prediction of Cell-Penetrating Peptides.. Int J Pept Res Ther.

[7] Hansen M, Kilk K, Langel U (2008). Predicting cell-penetrating peptides.. Adv Drug Deliv Rev.

[8] Dobchev DA, Mager I, Tulp I, Karelson G, Tamm T (2010). Prediction of Cell-Penetrating Peptides Using Artificial Neural Networks.. Current Computer Aided Drug Des.

[9] Provost F (2000). Machine Learning from Imbalanced Data Sets 101.. AAAI Workshop on Learning from Imbalaced Data Sets.

[10] Kersey PJ, Duarte J, Williams A, Karavidopoulou Y, Birney E (2004). The International Protein Index: an integrated database for proteomics experiments.. Proteomics.

[11] Anaspec I, Anaspec I (2010). Cell Permeable Peptides (CPP) / Drug Delivery Peptides.. Anaspec's Catalog Listing of Cell Permeable Peptides (CPP).

[12] Lopez F, Torres M, Batista B, Perez J, Moreno-Vega J (2006). Solving feature subset selection problem by a Parallel Scatter Search.. Eur J Oper Res.

[13] Platt J, Scholkopf B, Burges C, Smola A (1999). Fast Training of Support Vector Machines using Sequential Minimal Optimization.. Advances in Kernel Methods: Support Vector Learning.

[14] Ustun B, Melssen W, Buydens L (2005). Facilitating the application of Support Vector Regression by using a universal Pearson VII function based kernel.. Chemometrics and Intelligent Laboratory Systems.

[15] Soomets U, Lindgren M, Gallet X, Hallbrink M, Elmquist A (2000). Deletion analogues of transportan.. Biochim Biophys Acta.

[16] Liu H, Li J, Wong L (2002). A Comparative Study on Feature Selection and Classification Methods Using Gene Expression Profiles and Proteomic Patterns.. Genome Informatics.

[17] Hall M, Frank E, Holmes G, Pfahringer B, Reutemann P (2009). The WEKA Data Mining Software: An Update.. SIGKDD Explorations.

[18] Frankel AD, Pabo CO (1988). Cellular uptake of the tat protein from human immunodeficiency virus.. Cell.

[19] Derossi D, Joliot AH, Chassaing G, Prochiantz A (1994). The third helix of the Antennapedia homeodomain translocates through biological membranes.. J Biol Chem.

[20] Morris MC, Depollier J, Mery J, Heitz F, Divita G (2001). A peptide carrier for the delivery of biologically active proteins into mammalian cells.. Nat Biotechnol.

[21] Schumacher D, Tischer BK, Teifke JP, Wink K, Osterrieder N (2002). Generation of a permanent cell line that supports efficient growth of Marek's disease virus (MDV) by constitutive expression of MDV glycoprotein E.. J Gen Virol.

[22] Sanders WS, Bridges SM, McCarthy FM, Nanduri B, Burgess SC (2007). Prediction of peptides observable by mass spectrometry applied at the experimental set level.. BMC Bioinformatics.

[23] Kawashima S, Ogata H, Kanehisa M (1999). AAindex: Amino Acid Index Database.. Nucleic Acids Res.

[24] Gulaboski R, Scholz F (2003). Lipophilicity of Peptide Anions: An Experimental Data Set for Lipophilicity Calculations.. J Phys Chem B.

[25] Mitaku S, Hirokawa T, Tsuji T (2002). Amphiphilicity index of polar amino acids as an aid in the characterization of amino acid preference at membrane-water interfaces.. Bioinformatics.

[26] Sen TZ, Jernigan RL, Garnier J, Kloczkowski A (2005). GOR V server for protein secondary structure prediction.. Bioinformatics.

